# Rectal Sleeve for Fecal Diversion in the Management of a Sacrococcygeal Stage IV Pressure Ulcer in a Dementia Patient: A Non-surgical Alternative to Colostomy

**DOI:** 10.7759/cureus.93868

**Published:** 2025-10-05

**Authors:** Pedro Pereira, João Andrade, Helena Costa, Filipe Neves, Augusto Lourenço

**Affiliations:** 1 General Surgery, Guarda Local Health Unit, Guarda, PRT

**Keywords:** fecal diversion, non-surgical management, pressure ulcer, rectal sleeve, sacrococcygeal wound

## Abstract

Pressure ulcers are a serious and frequent complication in patients with moderate-to-severe limitations in their mobility. Advanced sacral ulcers are particularly susceptible to fecal contamination, which has a negative impact on wound healing. Surgical colostomy is often considered for fecal diversion but carries significant risks. This case presents an innovative, minimally invasive alternative using a rectal sleeve. We report the case of a 67-year-old Caucasian male with Alzheimer’s dementia, who presented with a chronic, stage IV sacrococcygeal pressure ulcer measuring 20 × 15 cm. Surgical debridement was performed, followed by the application of a rectal sleeve for fecal diversion. Combined with nutritional optimization and daily wound care, the ulcer showed marked improvement without the need for any surgical intervention. This case highlights the successful use of a rectal sleeve as a non-surgical alternative to colostomy in managing advanced sacral pressure ulcers. The technique allowed effective wound healing and avoided the risks associated with surgical fecal diversion. Further research may validate its broader clinical use.

## Introduction

Pressure ulcers are a well-known complication from lack of mobility, particularly in patients with dementia, with major clinical and socioeconomic implications [1]. These ulcers are often caused by prolonged pressure over bony prominences and exacerbated by factors such as malnutrition, anemia, hypoalbuminemia, and poor tissue perfusion [1,2].

Stage IV ulcers in the sacrococcygeal region are particularly challenging due to frequent contamination from feces [1]. Although colostomy, the surgical creation of an opening (stoma) in the abdominal wall to divert the fecal stream, is often employed as a fecal diversion strategy, its invasive nature and perioperative risks make it unsuitable or refused by some patients [3]. Recent studies have highlighted that patients with hypoalbuminemia and comorbidities are at higher risk of postoperative complications and mortality following colostomy, emphasizing the need for careful patient selection [4,5]. Additionally, even when fecal diversion is achieved, persistent contamination can still occur, potentially delaying healing [6]. This report describes a successful alternative approach using a rectal sleeve, offering effective fecal diversion without surgery.

## Case presentation

A 67-year-old white male with dementia presented with a stage IV sacrococcygeal pressure ulcer. The ulcer had been present for several months and measured 20 × 15 cm at presentation, with necrotic tissue covering the wound bed. The surrounding perilesional skin was macerated and inflamed, consistent with fecal contamination.

The patient had known risk factors for delayed healing, including hypoalbuminemia and mild normocytic, normochromic anemia. Nutritional assessment was performed, and a high-protein enteral diet supplemented with micronutrients was initiated, targeting 1.5-2.0 g/kg/day of protein. Serum albumin levels were monitored throughout the intervention.

**Figure 1:**
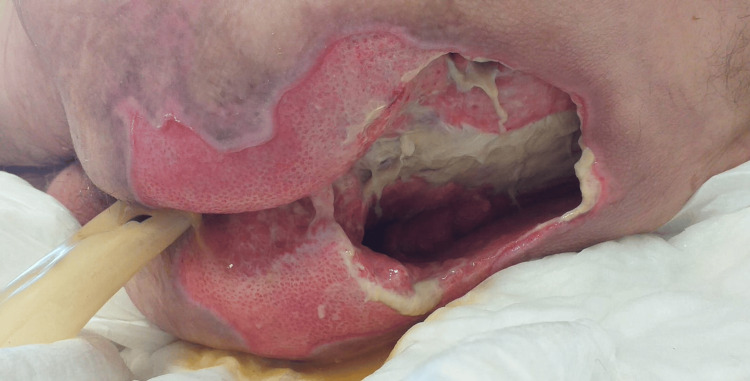
Sacrococcygeal pressure ulcer after initial debridement and application of a rectal sleeve.

Surgical debridement was undertaken to remove necrotic tissue and establish a viable wound base. To prevent further fecal contamination, a rectal sleeve (a silicone device inserted into the rectum to divert feces into a collection bag) was applied. Application of the device was performed according to the instructions provided by the manufacturer in the product packaging (Figure 1). The device was monitored daily for tolerance, including agitation (using the Agitated Behavior Scale), skin integrity, and signs of discomfort. Behavioral management strategies were applied without the use of physical restraints.

Daily dressing changes were performed using products that promoted a moist wound environment and autolytic debridement. Wound measurements were taken at defined intervals to track healing progression.

**Figure 2:**
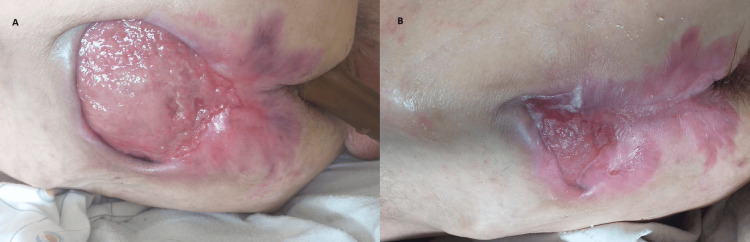
Evolution of the wound bed healing overtime. (A) Pressure ulcer after 20 days of application of the rectal sleeve. (B) Pressure ulcer after 30 days of application of the rectal sleeve.

Over the following four weeks, progressive wound improvement was observed. The ulcer gradually contracted, measuring 7 × 4 cm by the end of this period and sufficiently distant from the anal region to allow removal of the rectal sleeve. Healthy granulation tissue had formed, and epithelialization was advancing (Figure 2). No adverse events were associated with the rectal sleeve. The patient’s favorable clinical course precluded the need for surgical colostomy, and reconstructive surgery was deemed unnecessary due to successful conservative healing. Upon discharge, the patient had regained some autonomy; whereas he had been completely bedridden on admission, he was now able to ambulate with the support of caregivers and perform certain activities independently, such as feeding himself. By 50 days from the initiation of treatment, the pressure ulcer had achieved complete epithelialization and full closure, indicating successful resolution.

## Discussion

Pressure ulcers are associated with mobility disorders and poor nutritional and vascular status. Risk can be evaluated using tools such as the Braden Risk Assessment Scale and the Jackson-Cubbin Scale [7]. In trauma settings, higher Injury Severity Scores (ISS ≥25) have paradoxically been associated with a lower incidence of pressure ulcers, possibly due to increased monitoring [2].

Nutrition plays a vital role in wound healing. Collagen synthesis, immune response, and epithelial resurfacing are energy- and nutrient-dependent processes. While the importance of nutrition is established, high-quality clinical trials remain limited [7,8].

Wound care protocols emphasize moisture balance, infection control, and avoidance of further trauma. Creating a healing-favorable environment includes using dressings that promote autolytic debridement and protect perilesional skin [7-9].

In sacral ulcers, fecal contamination is a major setback to healing. Colostomy is often proposed, yet it presents challenges in high-risk patients [2]. Studies have shown that diverting colostomy may improve wound environment and quality of life in some patients, but the procedure carries a significant risk of complications, including stoma-related adverse events and delayed wound healing, particularly in patients with multiple comorbidities or previous abdominal surgeries [4-6,10]. The rectal sleeve provides an effective, non-invasive alternative for fecal diversion, assuring a clean wound environment while minimizing risk. This approach may benefit the most those patients to whom surgery is contraindicated because of prohibitive perioperative risk or who refused it [4,6,10]. It may be particularly useful in resource-limited settings.

## Conclusions

This case report describes an innovative, minimally invasive technique for fecal diversion using a rectal sleeve in a patient with a complex sacrococcygeal pressure ulcer. The approach was safe, well-tolerated, and allowed complete pressure ulcer healing, avoiding surgical intervention. Hence, rectal sleeves may become a viable and more usual option in managing pressure ulcers in high-risk or non-surgical candidates. Making the technique widespread could reduce complications, hospital length of stay, and improve the quality of life in immobilized patients. Further prospective studies are needed to validate this approach in larger cohorts.
